# Sensors Manufacturing on a 3D-Printed Transwell-Based Hybrid Organ-on-a-Chip for Non-Invasive Real-Time Biological Barrier Resistance Monitoring

**DOI:** 10.1021/acsbiomaterials.5c00923

**Published:** 2025-11-04

**Authors:** Simone Perottoni, Alessandro Bonacina, Ruben Dell’Oro, Lucia Boeri, Francesca Donnaloja, Luca Magagnin, Paola Petrini, Diego Albani, Carmen Giordano

**Affiliations:** † Department of Chemistry, Materials and Chemical Engineering “Giulio Natta”, 18981 Politecnico di Milano, Milan 20133, Italy; ‡ Department of Neuroscience, 9361 Istituto di Ricerche Farmacologiche Mario Negri IRCCS, Milan 20156, Italy

**Keywords:** TEER, organ-on-a-chip, bioreactor, 3D-printing, barrier

## Abstract

Biological barriers in our body are dedicated to maintaining systemic homeostasis by enabling selective transport, the dysregulation of which plays a pathogenic role in several diseases. Transendothelial/epithelial electrical resistance (TEER) analysis is conventionally used to assess the barrier permeability. Engineering TEER setups within advanced in vitro models has become essential for acquiring real-time information on barrier integrity inside organ-on-a-chip (OOC) devices. However, current approaches often involve multiple assembly steps, require external access to the culture chamber, rely on cumbersome systems, or provide highly localized measurements that do not represent the entire surface of the tissue under examination. Specifically, in Transwell-based OOC systems, continuous and nondisruptive monitoring is often precluded by the need to disassemble the system for TEER measurement. To address these limitations, we combined conventional technology for patterning thin conductive films with low-cost, benchtop 3D printing to integrate planar electrodes within a Transwell-based hybrid OOC system. Our integrated TEER electrode device, ITE-MINERVA, can be easily employed with commercial measurement instruments, representing a useful tool for real-time, in situ detection of barrier functionality, potentially altered by drug toxicity and absorption processes, on Transwell-based OOCs, both in single- and multiorgan configurations, noninvasively and without stopping the experiment. In this work, we present the design and fabrication strategy of our innovative setup and its assessment in recording real-time TEER modulation on a Caco-2 cell-based in vitro gut epithelial barrier model.

## Graphical abstract

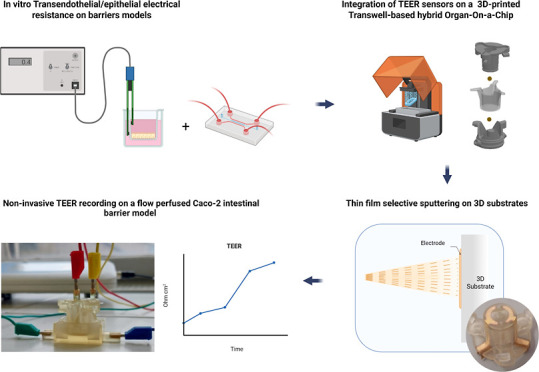

## Introduction

Biological barriers are fundamental to maintaining systemic homeostasis and mediating organ-to-organ crosstalk. Examples include the intestinal epithelium and the blood–brain barrier (BBB), both of which play crucial roles in regulating the selective passage of molecules. Alterations in their permeability have been implicated in the pathogenesis of various diseases.^1–3^ Therefore, accurately and reliably assessing barrier integrity in vitro is essential to ensure informative experimental outcomes. Transendothelial/epithelial electrical resistance (TEER) analysis is a nondestructive assay that indirectly evaluates the integrity of a biological barrier by measuring the ionic electrical resistance across the tissue. This technique enables real-time monitoring of both live cell dynamics throughout different physiological stages and the impact of external stimuli, such as toxins or drug compounds, on barrier function.^1^ TEER is particularly sensitive to changes in barrier tightness compared to molecular tracer assays and remains a widely employed method for characterizing the response of biological barriers to drug absorption, toxicity, and transport in several fields, including cancer and neuroscience research. It represents a key parameter in preclinical research, supporting the investigation of disease mechanisms and the identification of potential drug targets.^4^ Commercially available instruments for TEER measurement, such as EVOM or Millicell-ERS voltohmmeters,^1^ generally exploit ‘chopstick’ electrodes across Transwell cell culture inserts in the so-called tetrapolar (four-points) measurement approach. This method, which employs a pair of current-injecting electrodes and a pair of voltage-measuring electrodes, is widely recognized for reducing charging effects on both the cell layer and the electrodes.^5^ The recorded resistance includes contributions from the cellular layer, the porous membrane, the culture medium, and the medium/electrode interface. Beyond these basic setups, several commercially available multiwell systems, such as Locsense,^6^ xCELLigence,^7^ AKITA,^8^ and CellZscope,^9^ offer integrated, real-time TEER monitoring with embedded electrodes and automated recording capabilities. These platforms enable continuous, nondisruptive measurements across multiple inserts simultaneously, addressing some limitations of conventional chopstick-based approaches. In parallel, to meet the demands of more physiologically relevant models, research groups have been developing new strategies to custom engineer TEER setups specifically for organ-on-a-chip (OOC) devices.^10^ Indeed, conventional TEER measurement setups are often incompatible with most OOC systems due to their custom designs and small sizes. Various OOCs have successfully embedded electrodes and wiring for real-time, nondisruptive TEER monitoring,^11^ including solutions based on thin film deposition with soft lithography in microfluidic devices,^12^ patterned conductive traces on glasses or membranes subsequently assembled in the devices,^13,14^ the insertion of conductive wires through the chip walls,^15^ or the external insertion of conventional measuring probes like “chopstick” electrodes.^16^ All these strategies allow us to record both resistance and impedance on tissues cultured inside microphysiological system devices. However, current approaches often involve multiple assembly steps,^14^ require external access to the culture chamber thus introducing manipulations and risk of contamination,^17^ rely on cumbersome systems,^18^ or provide highly localized measurements^19^ that fail to cover the entire surface of the tissue under examination. Specifically, in Transwell-based OOC systems, continuous and nondisruptive monitoring is often precluded by the need to open the system^20^ or to disassemble the device in order to transfer the inserts into conventional multiwell plates for TEER measurement,^21,22^ thus interrupting the experiment. To overcome these limitations, we combined conventional thin conductive film patterning technology with low-cost, benchtop 3D printing for the integration of planar electrodes into a 3D-printed hybrid OOC^23,24^ hosting Transwell-like inserts for advanced in vitro modeling of biological barriers. We applied sputtering Physical Vapor Deposition (PVD), a widely used technique for depositing thin films on flat substrates, to the complex 3D geometry of our bioreactor by exploiting 3D-printed masks to generate nanometer-thick conductive traces on the device walls. This sensing approach on a complex 3D-printed fluidic technology ensures that the electrodes do not interfere with flow direction or cell culture procedures, allowing the electrical signal to be transmitted outside the device without the need for wires to cross the bioreactor. Our integrated TEER electrode device, the ITE-MINERVA (ITE-M), serves as a valuable tool for detecting barrier functionality, potentially altered by drug toxicity and absorption processes, on Transwell-based OOCs. Furthermore, thanks to its modular design, this sensorized unit can be incorporated into existing multiorgan in vitro platforms,^25^ enabling real-time monitoring of barrier models such as the intestinal epithelium and the BBB in response to pathological conditions, thereby enhancing outcome reliability and system ergonomics. In this work, we present the design and fabrication strategy of the ITE-M device and demonstrate its application in recording real-time TEER modulation on a Caco-2 cell-based gut epithelium barrier in vitro model.

## Experimental Methods

### Numerical Analysis

A computational analysis of the electrical current distribution was developed in COMSOL Multiphysics 6.0 (COMSOL Inc., Burlington, MA, USA). The aim of the numerical simulations was to identify the optimal electrode configuration to achieve a homogeneous current density across the cell layer on the permeable membrane and to compare it with the conventional TEER measurement setup. A discrete triangle element mesh was built by exploiting the “physics-controlled” method with an average side dimension of 0.2 mm in the region of interest. The “electric current” physics was used to simulate electrical current distribution through the system, and each domain of the geometry was associated with a material property (Table 1). Boundary conditions included (i) a ground domain at one of the ‘current injecting’ electrodes; (ii) a 10 μA current injection at the other “current injecting” electrode; (iii) zero electric potential [V] as an initial condition. No condition was set to the ‘potential recording’ electrode couple. The sensitivity was then calculated at each point of the cellular layer as follows:^19^

$$s=((J_{1}\times J_{2})/{(I}^{2}))\times A^{2}$$

where *I* [A] is the magnitude of the applied current, *J*_1_ [A/m] and *J*_2_ [A/m] are the current densities across the cellular layer for the two pairs of electrodes, and *A* [m^2^] is the area of the porous membrane. *J*_1_ and *J*_2_ have been computed in two subsequent studies with “current injecting”/“potential recording” (*I*–*V*) electrodes coupling exchanged. Specifically, a cross-aligned four-electrode configuration is used, with the current (*I*_1_–*I*_2_) and voltage (*V*_1_–*V*_2_) electrode pairs placed along diagonally opposed axes, forming a cross-pattern between the apical and basolateral compartments, and a symmetric, vertically aligned four-electrode configuration, with the current and voltage electrode pairs (*I*_1_–*I*_2_ and *V*_1_–*V*_2_, respectively) positioned along two parallel vertical axes, each spanning the apical and basolateral chambers. Results were finally expressed in terms of color-coded plots of sensitivity distribution at the cell layer, maximum and minimum values, and uniformity [%] of sensitivity in the range ±10. Moreover, we computed the terminals resistance [Ω], calibrated the model with the experimental measurements, and plotted the data next to the experimental data, obtaining a predictive trend.^26^

**Table 1:** Summary of the Material Properties Attributed to Each Geometry Domain in the Numerical Simulation

physical domain	electric conductivity [S/m]	relative permittivity
electrodes	45.6 × 10^6^	6.9 (mit.edu)
cell culture medium	1.5 ^27^	78.54 ^28^
porous membrane (PET)	0.00375 ^27^	3.4 (epci.eu)
cellular layer	21.23 ^29^	58,340 ^30^

### Electrodes Manufacturing by Physical Vapor Deposition

ITE-M components were fabricated by stereolithography (SLA) 3D printing using a photosensitive resin (Elegoo standard translucent UV resin, 405 nm). After printing, the samples were washed with a 70% isopropyl alcohol solution for 10 min. The curing process was then completed by exposing the components to UV light for 30 s using a UV curing workstation (Photo Electronics, Povegliano Veronese, VR, Italia). Nanometric thick electrodes were fabricated on both glass coverslips and 3D-printed components by PVD with a Leybold-Heraeus LH Z400 system (Oerlikon Balzers, Switzerland) at PoliFAB, Politecnico di Milano. This system is equipped with a vacuum chamber that enables thin-film deposition of various materials onto components ranging from millimeters to centimeters in size. Prior to PVD sputtering, custom 3D-printed masks with the desired conductive trace geometries were fabricated for both the round-shaped glass slides and 3D resin components. The sputtering setup was optimized to ensure homogeneous deposition of nanometric conductive layers on nonplanar surfaces (Figure S1). A 100 nm layer of titanium was deposited as an adhesion layer, followed by a 50 nm layer of gold as the conductive layer on both sides of the glass coverslips. On the 3D-printed components, a 100 nm gold layer was deposited. Thanks to the nanometric thickness of the conductive coating, optical imaging remained possible through the sensorized areas of the glass slide.

### Custom TEER Measurement Setup

All the TEER measurements were carried out by using the EVOM3 (World Precision Instruments (WPI), Sarasota, FL, USA), which is one of the most widespread instruments for TEER measurements. To take measurements with the ITE-M system, we made use of an electrode adapter (WPI #3993) connected to the EVOM3. STX2 commercial chopstick electrodes were used as a control. To perform the measurements, the sensorized device was placed into a custom-made, sensorized holder designed to interface with the EVOM3 system, enabling stable and operator-independent data acquisition.

### Resistance Recording on Membranes with Different Porosities

We assembled three different ITE-M devices, each equipped with a ThinCert cell culture insert (Greiner Bio-One S.r.l., Milano, Italy), featuring a porosity of 0.0025 (pore diameter: 0.4 μm). The apical and basal flow chambers were filled with cell culture medium supplemented with 20% fetal bovine serum (FBS) at room temperature, and nine measurements were recorded for each device. Subsequently, the three devices were disassembled, and the same procedure was repeated by using cell culture inserts with porosities of 0.015 and 0.125.

### Resistance Recording in Solutions at Different NaCl Concentrations

To verify the system’s ability to distinguish solutions with different conductivities, we tested the ITE-M device using three NaCl solutions at concentrations of 0.01, 0.1, and 1 M. Three separate devices were assembled, each equipped with a ThinCert cell culture insert (Greiner Bio-One S.r.l., Milano, Italy) with a porosity of 0.0025 (pore diameter: 0.4 μm). The apical and basal flow chambers were filled with a 0.01 M NaCl solution, and three resistance measurements were recorded for each device. Following this, the systems were rinsed with distilled water, and the same procedure was repeated using 0.1 and 1 M NaCl solutions.

### Cell Culture

Caco-2 human intestinal epithelial cells (ATCC) were cultured in high-glucose Dulbecco’s modified Eagle’s medium (Gibco), supplemented with 20% of heat-inactivated FBS (Gibco), 2 mM l-glutamine (Euroclone), 100 units/mL of penicillin, and 100 μg/mL of streptomycin (Euroclone). Caco-2 cells between passages 30 and 40 were seeded at a density of 5 × 10^4^ cells/cm^2^ on Transwell-like inserts (Greiner Bio-One ThinCert) with a surface area of 1.1312 cm^2^, pore diameter of 0.4 μm, and pore density of 2 × 10^6^ pores/cm^2^ previously coated overnight with 30 μg/mL collagen (Sigma-Aldrich). All the culture inserts were maintained at 37 °C, 5% CO_2_, and relative humidity (RH) > 93%.

### Cytotoxicity and Nondestructive Cell Viability Assay

To assess the cytotoxicity of the materials used in the fabrication of the experimental ITE-M device prototypes, a colorimetric MTS assay was performed on Caco-2 cells (CellTiter 96 AQueous One Solution Cell Proliferation Assay, Promega). The aim was to evaluate the potential cytotoxic effects of both the electrode coatings and the resin components. Gold-sputtered glass coverslips, resin samples, and gold-sputtered resin samples were sterilized by immersion in 70% ethanol, followed by UV exposure. All samples were then placed individually into the wells of a 12-well plate containing 1 mL of complete cell culture medium and maintained at 37 °C, 5% CO_2_, and RH > 93%. Aliquots of 750 μL of medium were collected at days 1, 3, and 7, replacing the removed volume with fresh medium at each time point. Caco-2 cells were seeded into 96-well plates at a density of 50,000 cells/cm^2^. After 48 h, the frozen eluates were thawed, and 250 μL of each eluate was added to triplicate wells (three wells per condition) following removal of the existing medium. After 24 h of incubation with the eluates, the medium was replaced with a 1:10 v/v dilution of MTS reagent in fresh culture medium. Following 2 h of incubation, absorbance at 490 nm was measured using a multimode microplate reader (SPARK, TECAN).

To assess Caco-2 cell viability in 3D culture conditions, a nondestructive viability assay was performed using Alamar Blue dye (Geno Technology Inc., USA). Cell samples were incubated with 300 μL of a 10% Alamar Blue solution, prepared in complete cell culture medium supplemented with 20% FBS, for 90 min at 37 °C, 5% CO_2_, and RH > 93%. Fluorescence intensity was measured using a SPARK multimode microplate reader (TECAN) with excitation and emission wavelengths set at 535 and 595 nm, respectively.

### Real-Time Detection of Intestinal Barrier Integrity during Flow Perfusion

We assembled each ITE-M device with a ThinCert cell culture insert (Greiner Bio-One S.r.l., Milano, Italia) with a porosity of 0.0025 (pore diameter 0.4 μm) previously seeded with Caco-2 cells. Each device was connected to two separate fluidic circuits filled with cell culture medium supplemented with 20% of FBS (one to the superior chamber and one to the inferior chamber), which were actuated by a peristaltic pump (G100-1L Peristaltic pump for incubator, Longer Precision Pump, UK) with a 30 μL/min flow rate. Details of the flow perfusion setup and its integration with the TEER measurement setup have been reported in Figure S2.

### Real-Time Detection of an Induced Leaky Gut Condition

Caco-2 cells were seeded on cell culture membranes and maintained in a multiwell plate for 2 weeks before being assembled into the sensorized devices. After 90 min, a 5 mM ethylenediaminetetracetic acid (EDTA) solution, prepared in complete cell culture medium supplemented with 20% of FBS, was injected into the apical chambers. The devices were maintained at 37 °C, 5% CO_2_, and RH > 93%. TEER values were recorded every 30 min.

### Assessment of the Effects of an Intestinal Mucus-Like Hydrogel on TEER

A mucus-like alginate-based hydrogel (Bac3Gel) was produced as previously described.^31^ The following day, six ITE-M devices were assembled using complete cell culture medium supplemented with 20% of FBS, and subsequently maintained at 37 °C, 5% CO_2_, and RH > 93%. TEER measurements were recorded at three time points over a 4 h period, with three readings per time point for each device. Control measurements using the hydrogel without cells were also performed to assess its contribution to the overall electrical resistance.

### Optical Microscopy and Immunofluorescent Analysis

In order to monitor the integrity of the cell layer during experiments, broad field microscopy was performed directly through the device with the inverted microscope DMi1 (Leica Microsystems) equipped with 5×, 10×, 20×, and 40× objectives. The ITE-M device basal chamber includes a standard microscopy glass coverslip positioned 500 μm below the cell culture membrane to ensure compatibility with both bright-field and fluorescence microscopy. This design was previously optimized to guarantee full optical accessibility for real-time monitoring of cellular morphology.^23,24^

After removing the samples from the ITE-M device, they were rinsed twice with PBS with Ca^2+^ and Mg^2+^ for 5 min and fixed for 40 min in paraformaldehyde (4% PAF). Samples were then washed three times for 5 min in PBS. To block nonspecific binding of antibodies, 300 μL of blocking solution, composed of 0.25% Triton-X-100, 4% normal goat serum (NGS) in PBS, was added to each sample for 1 h at room temperature. Samples were incubated overnight at 4 °C with primary antibodies diluted in PBS with 0.25% Triton-X-100 and NGS 1%. To visualize occludin, we used mouse antioccludin monoclonal antibody (Invitrogen) diluted 1:100. The next day, samples were rinsed three times for 5 min in PBS and incubated with the Alexa Fluor 488 goat antimouse IgG secondary antibody (Jackson IR) diluted 1:750 at room temperature for 45 min. After three sample rinses (5 min in PBS), cell nuclei were labeled with Hoechst 33342 (Thermo Fisher) diluted 1:1000 for 10 min at room temperature. Finally, samples were washed with PBS and distilled water and mounted with 10 μL of MOWIOL 4–88 Reagent (Sigma-Aldrich, USA) on microscope slides. Fluorescence images were acquired with the confocal microscope (Fluoview FV10i, Olympus, Japan), equipped with a 60× water immersion objective, 1.2 numerical aperture, and 0.28 wide-field. Excitation/emission wavelengths of 473/490–590 nm and 352/455 nm were selected for occludin and Hoechst channels, respectively.

### Statistical Analysis

All statistical analyses were performed using GraphPad Prism (GraphPad Software, Inc.). Results are presented as the mean ± standard deviation (SD), and cell viability data are reported as percentages. To assess statistically significant differences: one-way ANOVA was used for comparisons among multiple experimental groups in the functional assessment assays (Figure 3) and cytotoxicity assay (Figure S4); one-way repeated measures ANOVA was applied to analyze TEER values recorded at different time points within the same experimental group (Figures 3 and 4); an unpaired *t*-test with Welch’s correction was employed for comparisons between the 2D and 3D experimental groups (Figure 4). A *P* value of <0.05 was considered significant: *P < 0.05, **P < 0.01, ***P < 0.001, and ****P < 0.0001.

## Results and Discussion

Among the various strategies for monitoring biological barriers in vitro,^32,33^ the most widely used method relies on measuring TEER across the cellular layer forming the barrier.^14^ In this work, we describe the development of a new sensorized, modular device for noninvasive, real-time TEER investigation, designed for use in advanced on-a-chip Transwell-based in vitro models. We introduce a novel fabrication approach that utilizes sputtering, a commonly employed PVD technique typically used to create conductive surfaces on microdevices, and apply it to complex, large-scale 3D-printed components, representing a novelty in the manufacturing process of sensorized bioreactors.

The design of the ITE-M device builds upon a previously developed system^23,24^ with the integration of two pairs of electrodes to enable tetrapolar TEER measurement, a configuration known to reduce charge polarization effects^18^ (Figure 1a,b). PVD sputtering allows the deposition of nanometric films of different materials onto components ranging from millimeter to centimeter in size, ensuring good adhesion to the substrate, precise control over film thickness and composition, and high uniformity.^34^ We customized the PVD process to selectively sputter nanometer-thick electrodes directly within the fluidic chambers (Figure 1c,d), using 3D-printed masks to draw 3D conductive pathways along the walls of the bioreactors (Figures S1 and S3). This configuration allows direct interfacing of the internal conductive layers with external recording instruments, eliminating the need for wires, pins, or access holes, commonly required in other sensorized OOC systems,^15,16^ thus reducing handling complexity and preserving sterility. Most sensorized devices reported in the literature rely on custom-made membranes for cell culture, as seen in the works of Chacón et al.^18^ and Rajasekaran et al.^13^ In contrast, for millifluidic devices using commercially available cell culture inserts,^35–37^ TEER measurement typically requires dismantling the bioreactor. To the best of our knowledge, our manufacturing strategy has produced the first sensorized Transwell-based bioreactor for real-time TEER monitoring. In this work, we focused on single-frequency TEER acquisition by interfacing our system with the EVOM3, a widespread commercial instrument for resistance recordings,^38^ as similarly done by Chapin et al.^39^ and Booth et al.^40^

**Figure 1:**
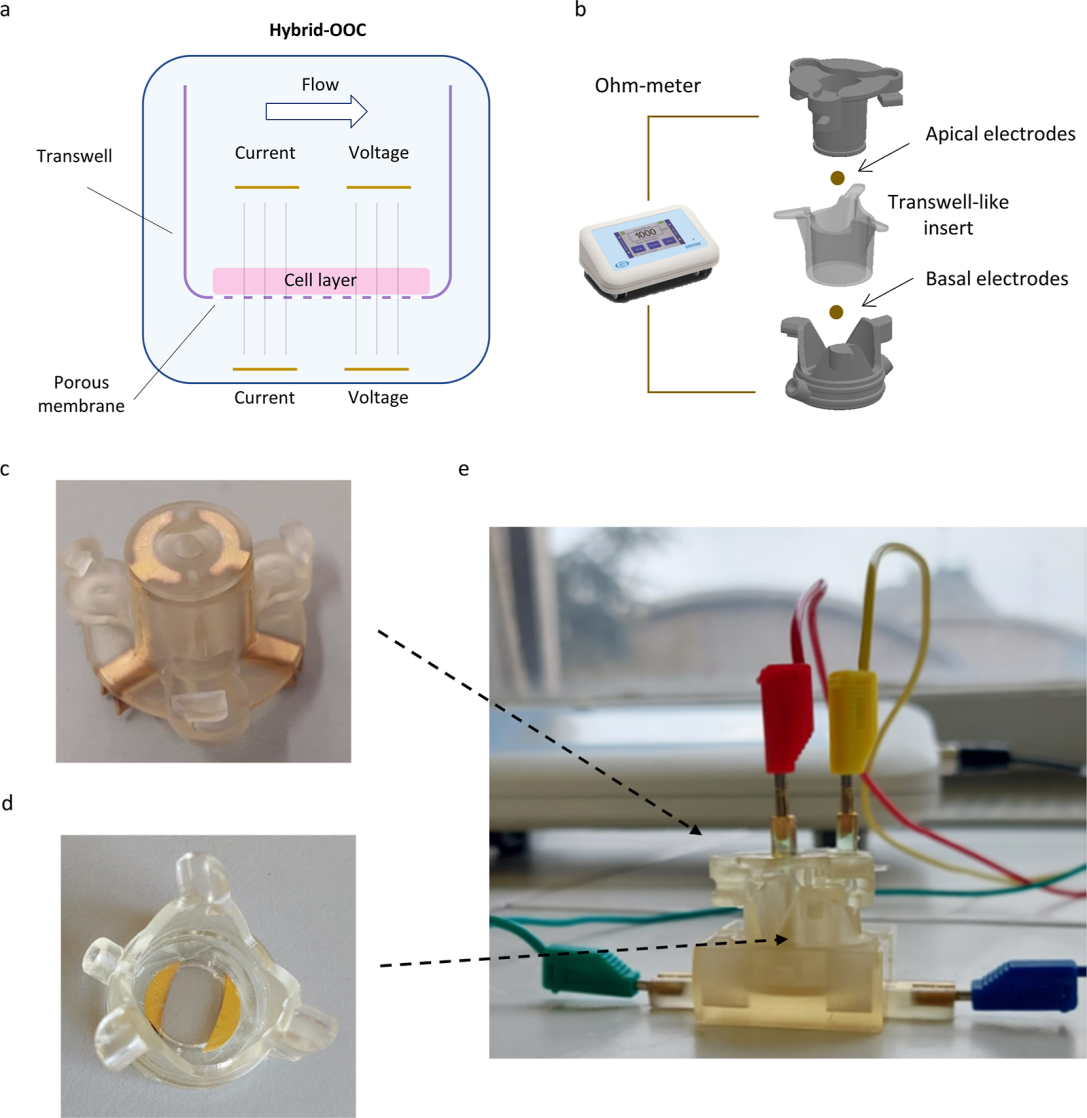
ITE-MINERVA device setup. (a) Schematic representation of the bicompartmental flow chamber of the bioreactor, showing the electrodes arranged in a tetrapolar measurement configuration. (b) Conceptual diagram illustrating the different components of the system. (c) Electrodes selectively deposited on the 3D-printed apical component. (d) Sensorized microscopy glass in the basal component. Both apical and basal parts feature specular semicircular electrodes as part of the same “symmetric” configuration. (e) Fully assembled sensorized platform in the recording configuration.

To tailor our TEER setup, we evaluated different electrode design configurations, which are known to influence measurement outcomes in custom-engineered OOCs.^27^ The numerical analysis of current density distribution revealed that sensitivity, a widely used parameter in the literature,^29,38^ depended on electrode size, position, and geometry (Figure 2). Our results confirmed that ITE-M electrodes with larger surface areas generated more uniform current distributions compared to conventional chopstick electrodes, in agreement with findings reported by Yeste et al.^29^ Indeed, the traditional chopstick electrode configuration produces a highly localized current excitation, where regions closest to the electrodes contribute disproportionately to the total resistance.^17^ Our model confirmed that sensitivity peaks occur near the electrode pairs, while sensitivity valleys appear in the regions between them,^29^ thereby explaining the low signal uniformity observed with both conventional chopstick electrodes and ITE-M punctual configuration. On the contrary, concentric and symmetrical electrode arrangements resulted in a more homogeneous current distribution, as the larger conductive areas cover a greater portion of the cell culture membrane. Furthermore, numerical analysis demonstrated that the symmetric vertically aligned current/voltage excitation configuration provides the most uniform current density across the cellular layer, regardless of electrode geometry or size.

**Figure 2:**
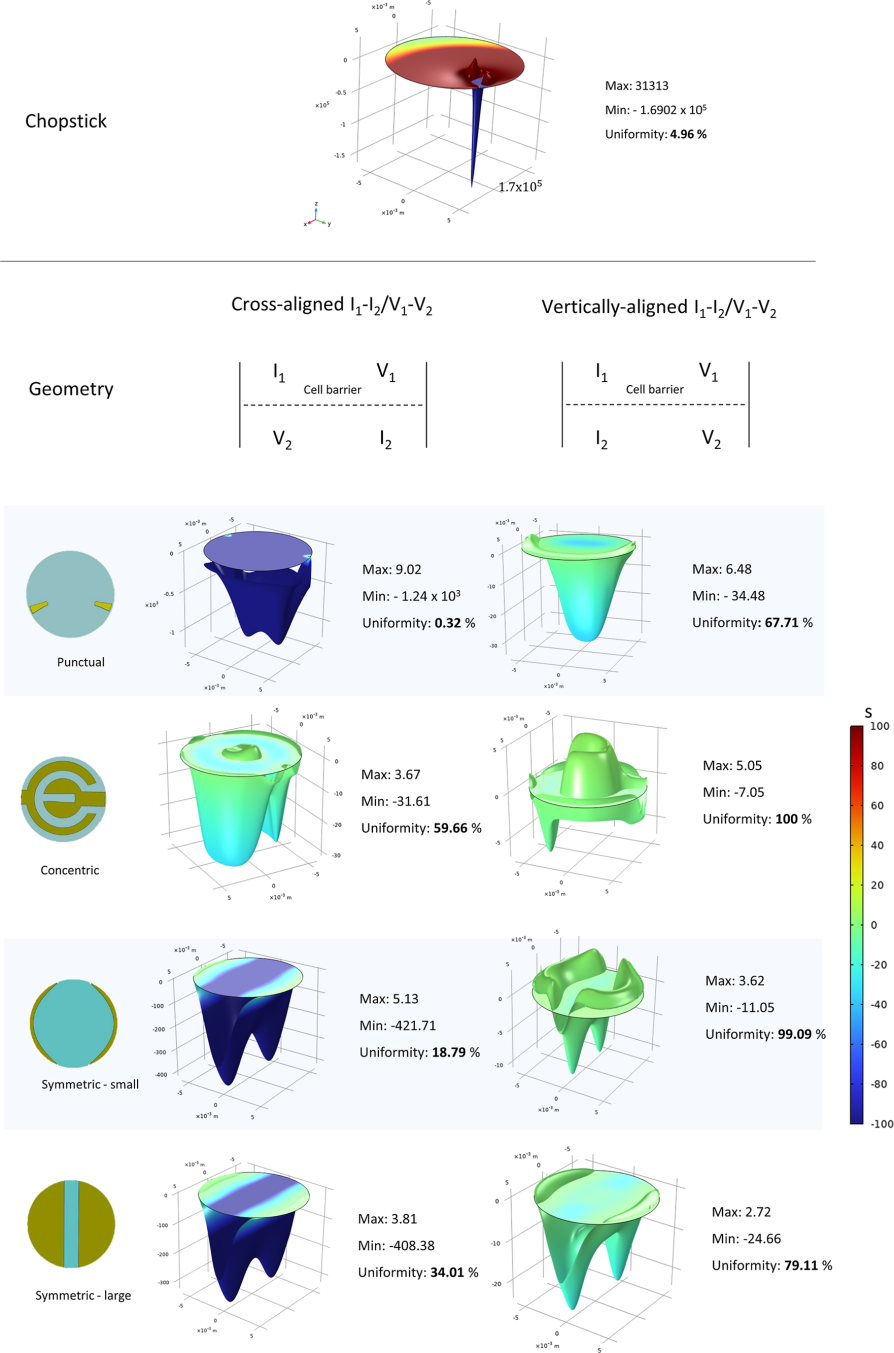
Color-coded 3D plots showing the sensitivity distribution on the cell layer both in the reference model chopstick measurement approach and in the ITE-M device at different electrode geometries and *I*_1_–*I*_2_/*V*_1_–*V*_2_ coupling configurations. The geometries depicted represent the same electrode configuration implemented on both the apical and basolateral components. Color scale bar range was maintained unvaried in all the plots for better comparison between the models.

To assess the ITE-M functionality, we experimentally tested the “symmetric” electrode configuration. The system successfully detected resistance values of a commercial permeable membrane with known porosity (Figure 3a), showing enhanced reproducibility and lower absolute resistance values compared to conventional chopstick electrodes. This is likely due to the increased electrode surface area, which typically leads to lower resistance readings. In contrast, using chopstick electrodes with relatively large membranes often results in overestimated TEER values, as previously reported.^1^ For comparison, TEER measurements on 0.4 μm pore-size membranes without cells (i.e., “naked” membranes), obtained using cellZscope, a commercial system with large concentric electrodes placed near the culture surface, yielded values of 41 ± 4 Ω cm^2^, which closely match our experimental data.^41^ Our observation was confirmed by resistance numerical analysis that suggests how position, geometry, and size in the two different configurations could dictate the overall resistance recorded value.

**Figure 3:**
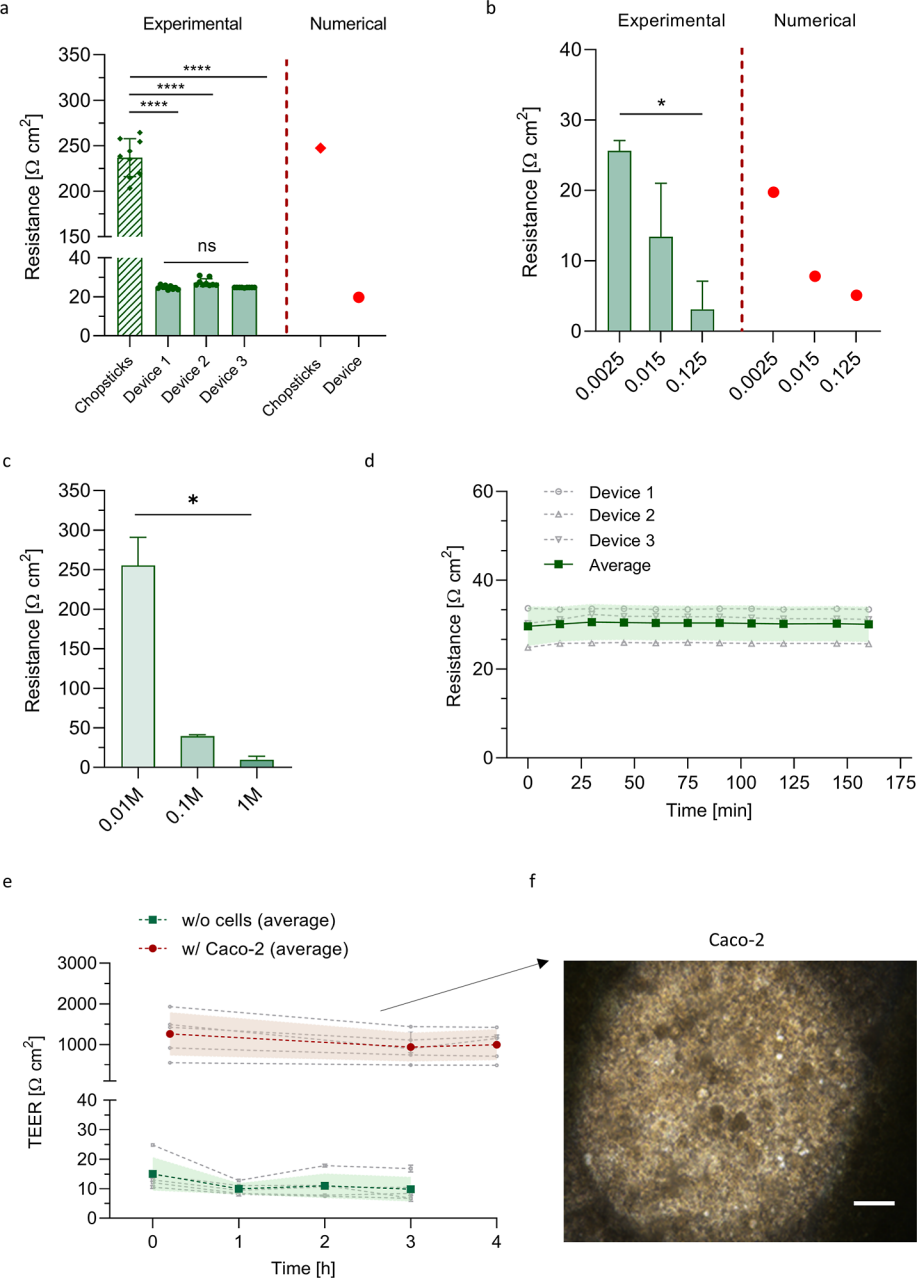
Functional assessment of the ITE-M device. (a) Experimental and numerical resistance comparison between ITE-M symmetrical electrode configuration and STX2 (chopsticks) electrodes; membrane porosity: 0.0025. ****: *p*-value <0.0001. (b) Functional assessment of TEER recordings on membranes with different porosities and (c) different NaCl concentrations, compared to numerical predictions. One-way ANOVA was used to detect statistical differences with *: *p*-value <0.05; *n* = 3. (d) Real-time monitoring in static and in (e) flow perfusion conditions without (green) and with (red) Caco-2 cells. Green and red diagrams refer to distinct experiments. *n* = 5. No statistical differences were detected by one-way repeated measures ANOVA test. (f) Representative optical microscopy image of Caco-2 cells cultured within the ITE-M device and imaged directly in the bioreactor, highlighting the in situ observation capability of the device. Scale bar: 300 μm.

We tested the measurement system’s capability to discriminate resistance changes in response to variations in systemic parameters such as membrane porosity and medium salt concentration. The ITE-M device was demonstrated to significantly discern different porosities of the commercial permeable membranes, which correspond to distinct resistance values. Specifically, resistance decreased as membrane porosity increased (Figure 3b). Additionally, the system recorded a consistent decrease in resistance with increasing solution conductivity (i.e., higher NaCl concentration), showing statistically significant differences (Figure 3c). This functional assessment is commonly reported in the literature,^27,42^ and the results were in agreement with those of Henry et al.^12^ and Cacopardo et al.,^14^ who observed a decrease in measured resistance by 1 order of magnitude when increasing the NaCl concentration from 0.01 to 0.1 M, which correspondingly increased the solution conductivity in their OOCs. Finally, the ITE-M device proved to be suitable for real-time resistance monitoring, without requiring removal from the cell culture incubator, under both static and flow perfusion conditions (Figure 3d,e). While several commercial and research-stage OOC platforms have been specifically designed for high-throughput in-incubator TEER monitoring,^6–9,43^ our solution represents a relevant improvement in the context of TEER measurements in pump-driven perfused OOC systems based on Transwell formats,^20,21,35,44^ as also evidenced by the absence of such systems in recent reviews in the field.^45,46^ The measured values varied in the range of a few Ω, likely due to temperature stabilization over time^17^ and the flow perfusion itself. In particular, the measurement system recorded real-time resistance changes with an average difference between the last and first time points of 0.49 Ω cm^2^ under static conditions and 2.66 Ω cm^2^ when flow perfusion (30 μL/min) was applied. Caco-2 TEER was recorded in time during flow perfusion, showing an average TEER around 1000 Ω cm^2^ throughout the experimental time (Figure 3f).

To evaluate the system’s performance in monitoring TEER changes in epithelial cell layers, we employed the ITE-M device using a Caco-2 cell model, following confirmation of its noncytotoxicity on the cells (Figure S4). Caco-2 cells spontaneously form a compact biological barrier on the membrane of the cell culture insert^47^ and are widely used as an intestinal model to assess OOCs functionality.^13,48^ In particular, we employed two representative scenarios (i.e., EDTA exposure and 3D environment) known to influence barrier permeability in vitro and thus induce a change in TEER over time (Figure 4).

**Figure 4:**
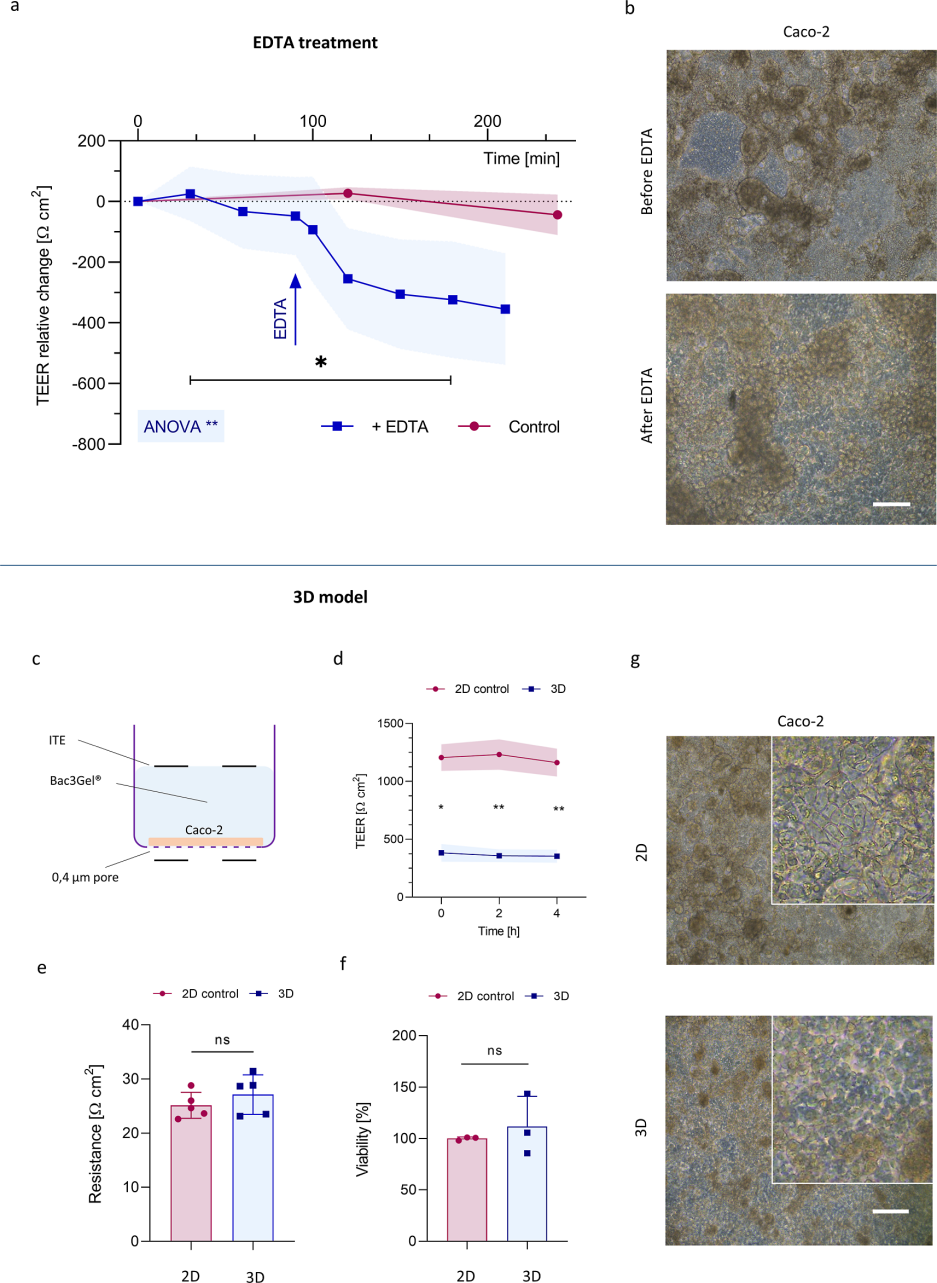
Real-time TEER measurement on Caco-2 epithelium with the ITE-M device. (a) TEER relative change: untreated control group (red); EDTA-treated group (blue). Red and blued diagrams refer to distinct experiments. One-way repeated measures ANOVA was used to detect statistical differences with *: *p*-value <0.05, **: *p*-value <0.001; *n* = 3. (b) Optical microscopy of Caco-2 cells inside ITE-M device before and after EDTA solution treatment. Scale bar: 100 μm. (c) Evaluation of the integration of Bac3Gel on Caco-2 cells by ITE-M measurements: (d) TEER in time after 24 h of 3D culture. *t*-test with Welch’s correction was used to detect statistical differences; **: p-value <0.01; *n* = 3. (e) Resistance values measured in plastic inserts with culture medium only (2D, red) and inserts with Bac3Gel and culture medium (3D, blue), both without cells. *t*-test with Welch’s correction was used to detect statistical differences; ns: not significant; *n* = 5. (f) Results of Alamar Blue cell viability assay on Caco-2 cells. *t*-test with Welch’s correction was used to detect statistical differences; ns: not significant; *n* = 3. (g) Optical microscopy of Caco-2 cells cultured in 2D and 3D. Scale bar: 100 μm. Enlarged picture on upper right corner is a software assisted enlargement of the same image.

A decrease in TEER was recorded immediately after EDTA exposure, confirming its known effect on barrier integrity, as previously reported^49^ (Figure 4a). TEER was measured every 30 min in devices hosting Caco-2 cell layers. A 5 mM EDTA solution was injected inside the devices at a time point of 90 min. A rapid average decrease of 35.6% (SD ± 3.6) in TEER was detected at 130 min. The signal then gradually declined over the next three time points, reaching a final drop of 62.1% (SD ± 1.1) at 210 min compared with the initial value. In contrast, a control experiment without EDTA exposure reported stable TEER readings throughout. Real-time optical microscopy was used alongside TEER monitoring to observe morphological changes in Caco-2 cells following EDTA treatment. The cells appeared rounded compared to their morphology before the stimulus was applied (Figure 4b), a characteristic feature of a leaky gut in vitro model, where tight junctions are loosened.^49^ In particular, the presence of 3D villi-like structures in a complex layer organization can be recognized in the control group,^23^ while it is not present anymore in the treated samples.

TEER values are generally lower in Caco-2 cells grown with 3D matrices that mimic the extracellular matrix compared to conventional 2D models. These 3D systems are considered to better reflect physiological conditions.^50^ To evaluate the performance of our ITE-M prototype in monitoring barrier integrity in such advanced models, we tested it in the evaluation of the effects of a 3D bioinspired intestinal mucus model on Caco-2 cells (Figure 4c). The intestinal mucus is a biological hydrogel that provides a suitable environment for bacterial colonization while preventing epithelial infection and promoting gut homeostasis.^51^ A bioinspired in vitro mucus model (Bac3Gel) has been developed^32^ to mimic the in vivo viscoelastic properties of native mucus, thereby offering a more physiological environment to intestinal cell cultures. We applied this alginate-based hydrogel mucus model on Caco-2 cells and assessed its effect on the intestinal barrier permeability. The ITE-M device recorded lower TEER values on Caco-2 cells cultured in the presence of the 3D mucus model (Figure 4d) compared to control conditions. A significant TEER gap between the two groups was already evident at time point zero (762.3 Ω cm^2^) and persisted for up to 4 h. No significant contribution from the alginate-based hydrogel alone to the overall resistance was detected (Figure 4e). Although the presence of the hydrogel did not compromise cell viability (Figure 4f), a shift in the cell morphology was observed (Figure 4g). However, immunofluorescence analysis of tight junction expression (Figure S5) qualitatively confirmed the presence of intact Caco-2 monolayers following ITE-M measurements, showing occluding expression both in the presence and without the presence of the 3D mucus model. The present results demonstrate the application of ITE-M devices for evaluating the integration of a 3D bioinspired mucus model on intestinal epithelial cells, showing its suitability for real-time detection of barrier integrity in advanced 3D in vitro models. Specifically, TEER analysis revealed the potential modulatory effects of the mucus model on barrier permeability, confirming the TEER reduction induced by the presence of a hydrogel-based matrix, as already observed in literature with chitosan-coated alginate microspheres or collagen I hydrogels.^52,53^

Overall, although our system was shown to provide relatively consistent measurements, the absolute TEER values in the cellular models still require in-depth characterization. Srinivasan and colleagues compiled both in vivo and in vitro TEER values recorded on biological barriers,^1^ reporting in vivo TEER for the colon between 300 and 400 Ω cm^2^. In vitro TEER values are 10 orders of magnitude higher and vary widely depending on the cell model and measurement system, with differences of several hundred Ω cm^2^. For example, TEER measurements in intestinal models using Caco-2 cells range in the literature from 250 to 2400 Ω cm^2^. Notably, Feighery et al. recorded a TEER of 763 Ω cm^2^ with a SD of ±287 with the Endohm chamber.^54^ This variation of hundreds of Ω cm^2^ could explain what we also observed in the measurements with the ITE-M system. Moreover, it is worth mentioning that variability in TEER measurements on Caco-2 cell-based models is often attributed to heterogeneity within cell subpopulations and differences in cellular differentiation states.^55–57^ However, the future implementation of impedance-based spectroscopy could enhance the performance of our system. The four electrical outputs of the ITE-M device can be connected to an impedance meter, enabling frequency-resolved impedance analysis and more robust modeling of the barrier’s electrical properties, taking into account both the resistive and capacitive contributions of the cellular monolayer.^58^

Overall, our work gives proof of the employability of the system in conventional TEER assays, with the possibility to take real-time TEER measurements, which is also one of the best advantages compared to existing Transwell-based hybrid–OOCs^59,60^ lacking integrated electrodes, in which TEER measurement represents an end point assay.^58^ While this study focused on demonstrating the feasibility and functionality of the device using standard resins, the integration of certified biocompatible materials will be a key step in future iterations aimed at more widespread in vitro biological applications. Indeed, biocompatible resins compliant with the ISO 10993 standard^61–63^ are now available with sufficient resolution to meet the fabrication requirements of the ITE-M device. These materials represent a promising direction for scaling up our technology and extending its applicability to a broader range of cell models. In this scenario, our strategy enables the translation of the miniaturized sensing approach to a wide range of organ and tissue models and is readily adaptable to customized bioreactor configurations. The system is compatible with widely used bicompartmental barrier models such as gut-on-a-chip, BBB-on-a-chip, and kidney-on-a-chip, as well as more complex air-to-liquid interface (ALI) models, including lung-on-a-chip, skin-on-a-chip, and advanced gut-on-a-chip platforms.^4^ Notably, starting from the Caco-2 cell-based model validated in this study, which has also been shown in the literature to be suitable for coupling with ALI conditions to investigate a physiologically relevant human intestinal barrier,^64^ the ITE-M device would allow TEER measurements under ALI conditions by temporarily adding a small volume of apical medium to ensure proper electrode contact through the fluidic ports during the experiment.

The modularity of the ITE-M system supports its future implementation in multiorgan-on-a-chip platforms, which represent one of the current frontiers in in vitro disease modeling.^65^ Multiple ITE-M devices can be assembled in a plug-and-play manner into a dedicated holder with dimensions of a standard multiwell plate.^25^ This configuration can be integrated into existing platforms investigating systemic axes such as the microbiota-gut-brain axis,^66^ enabling the real-time assessment of barrier responses to specific treatments, including bacterial toxins,^31^ drugs,^25^ or circulating immune components known to modulate biological barrier permeability^67^ in a dynamic flow perfused environment, which has proven to favor epithelial polarization and maturation.^23^

Throughput, which currently represents one of the limitations of our approach, could be improved thanks to the presence of dedicated electrical connection pins in each device. These allow for routing signals into a multiplexer capable of managing multiple channels simultaneously. While there are existing commercial and research platforms offering high-throughput TEER measurements,^68^ the ITE-M system has the potential to achieve a throughput comparable to that of a 12-well plate. This allows for the simultaneous analysis of multiple samples with a sufficient cell number per condition, a key requirement in assays involving complex biological responses or higher biomass needs. Optimization of the electronic circuitry and sensorization masks will enable the development of a compact system with a footprint similar to standard multiwell plates, improving data acquisition efficiency and ensuring a balance between throughput, data quality, and operational costs. Moreover, the increase of the sampling frequency of the measurements, as successfully demonstrated by Jones et al., who performed recordings at 1 min intervals using their customized sensors, could enhance the system’s performance in resolving and accurately capturing dynamic barrier-modulating events, such as drug actions or toxic effects.^69^ A further limitation of the current approach concerns the scalability of the fabrication process. While sputtering has proven particularly suitable in this work for conformal deposition, enabling uniform coating on nonplanar surfaces, its upscaling will require careful optimization of machine loading efficiency and cost-effective production workflows. Nonetheless, additional low-cost fabrication strategies that do not require specialized cleanroom facilities may be explored, as suggested by recent literature. These include techniques such as screen printing of electrodes on thermoplastic OOC substrates^70^ and the use of custom flexible printed circuit boards.^4^

## Conclusion

The ITE-M system integrates functional TEER monitoring in a hybrid–OOC Transwell-based device via a conventional fabrication process adapted to complex 3D printing. This approach enables thin-film sensing while preserving optical accessibility, ensuring optimal fluidics, and maintaining ergonomic design for noninvasive, real-time monitoring, particularly suited for laboratories requiring customizable configurations. Overall, the ITE-M system proved TEER discrimination in advanced in vitro cell barriers while being compatible with widely used recording instruments, thus contributing to the development of alternative and more standardized approaches for sensor integration in millifluidic-scale OOC platforms.

## Supplementary Material



## Data Availability

All supporting data are available by contacting the corresponding author.

## Notes

Additional setup details, cytotoxicity, and immunostaining analysis (PDF)
