# Supplementary material for: Sensors Manufacturing on a 3D-Printed Transwell-Based Hybrid Organ-on-a-Chip for Non-Invasive Real-Time Biological Barrier Resistance Monitoring
Source: ACS Biomater Sci Eng. 2025 Nov 4;11(12):7570–81. doi: 10.1021/acsbiomaterials.5c00923 (PMC12690515; doi:10.1021/acsbiomaterials.5c00923)
Supplement: Supplementary file 1 [file ab5c00923_si_001.pdf]

Supplementary data for "Sensors Manufacturing on a 3D-Printed Transwell-Based Hybrid Organ-On-a-Chip for Non-Invasive Real-Time Biological Barrier Resistance Monitoring"

Simone Perottoni[1], *Alessandro Bonacina*[1], *Ruben Dell'Oro*[1], *Lucia Boeri*[1], *Francesca Donnaloja*[1], *Luca Magagnin*[1], *Paola Petrini*[1], *Diego Albani*[2] *and Carmen Giordano*[1]

1. Department of Chemistry, Materials and Chemical Engineering "Giulio Natta", Politecnico di Milano, 20133, Milan, Italy

2. Istituto di Ricerche Farmacologiche Mario Negri IRCCS, Department of Neuroscience, 20156, Milan, Italy

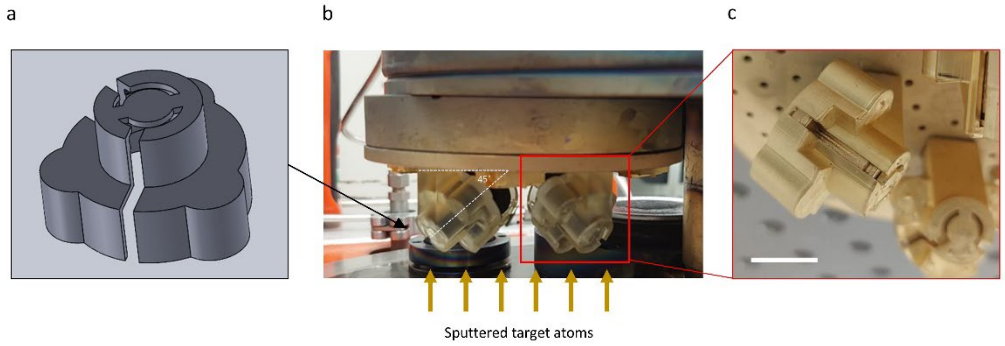

Figure S1. Details of the sputtering setup used in the Leybold-Heraeus LH Z400 system. a) CAD model of the 3D-printed mask designed for the selective deposition of conductive layers on the apical component of the ITE-M device. b) Samples mounted on the substrate holder of the sputtering system at 45° inclination to ensure uniform deposition of nanometric conductive layers on non-planar surfaces. c) Close-up view of the sputtered samples. Scale bar: 1 cm.

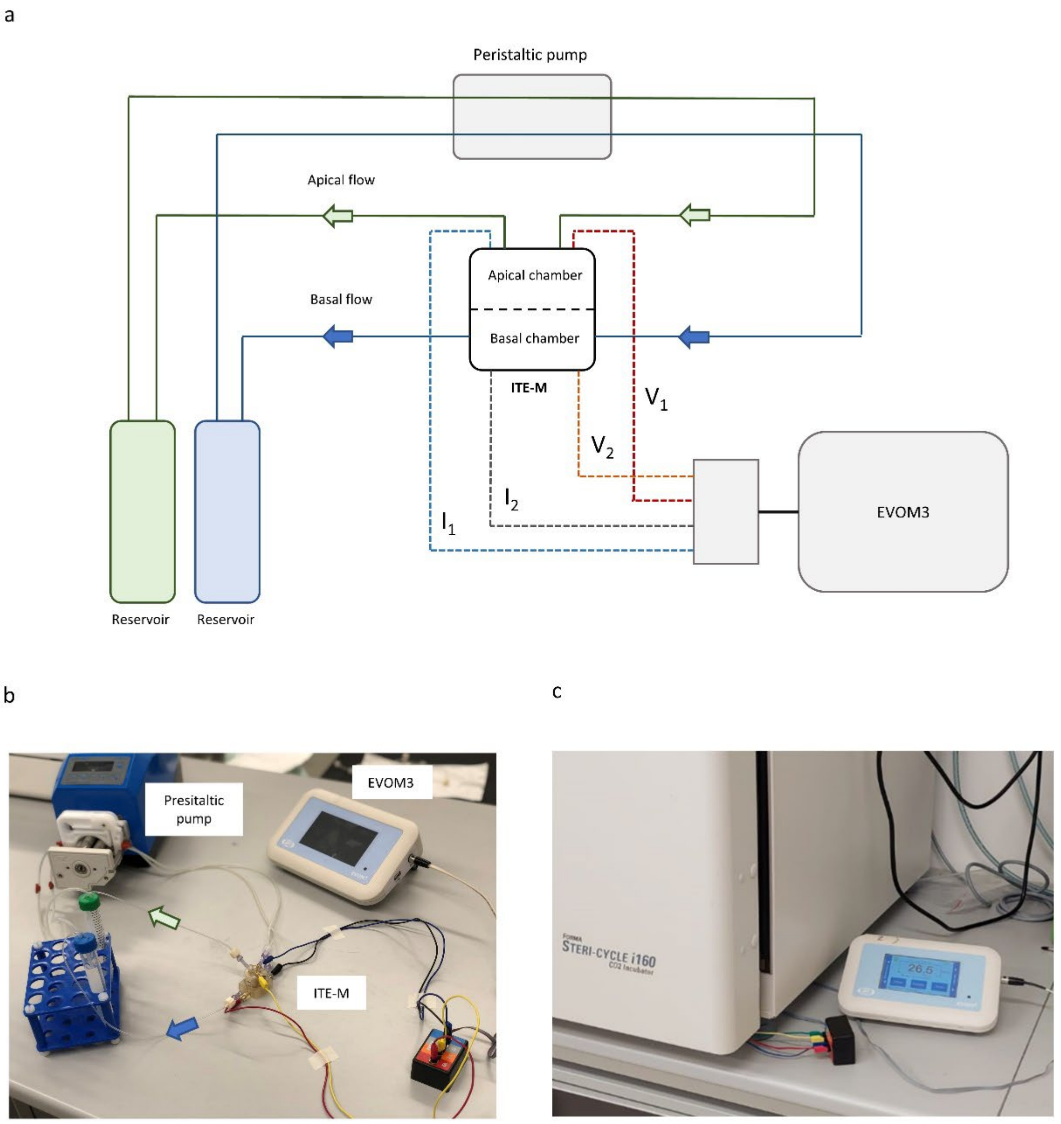

Figure S2. a) Schematic representation of the fluidic and electrical circuits. The apical and basal flow circuits are connected to a peristaltic pump and to their respective collection reservoirs. Current-injecting and voltage-measuring electrodes are connected via an adapter to the EVOM3 instrument. b) Real-time and c) in-incubator TEER measurement setup during flow perfusion of the ITE-M device.

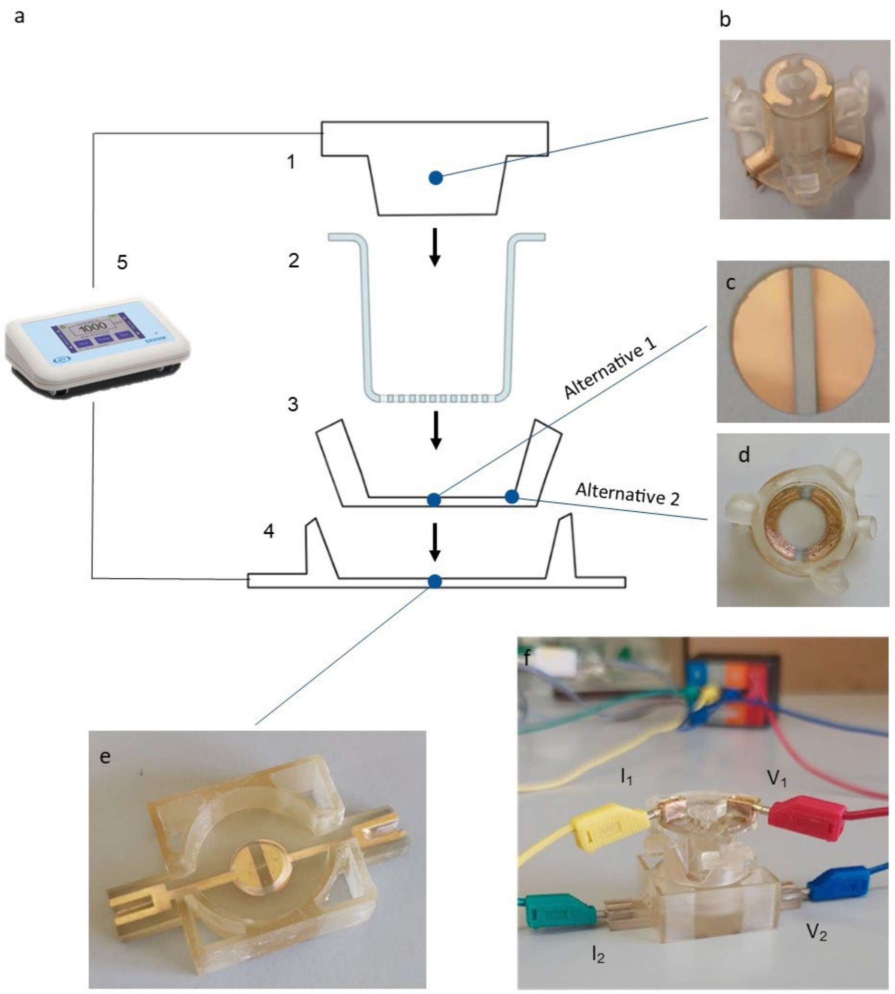

Figure S3. Detail of the assembly and sensorization of the ITE-M device. a) The different components of the device (1. Superior-apical part; 2. Cell culture insert; 3. Inferior-basal part; 4. Holder; 5. EVOM volt/ohm meter) are assembled through successive insertions. b) The upper part houses the electrodes I1 and V1, while the inferior part contains electrodes I2 and V2, which can be fabricated c) on a microscope slide or d) directly on the walls of the components. e) The measurement holder houses a conductive track that makes contacts with the electrodes of the inferior part. f) In this way, the electrical circuit is closed and stably connected to the measuring instrument, without the need of manual intervention on the system.

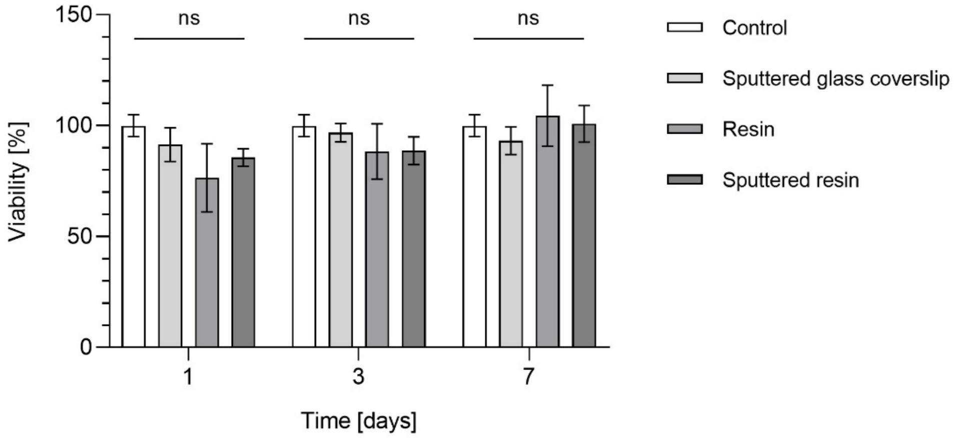

Figure S4. Results of the MTS cytotoxicity assay performed on Caco-2 cells using eluates from the ITE-M device components/materials. The x-axis indicates the time point at which the eluates were collected (1, 3, and 7 days). One-way ANOVA test revealed no statistically significant differences in cell viability among the groups. ns: not significant.

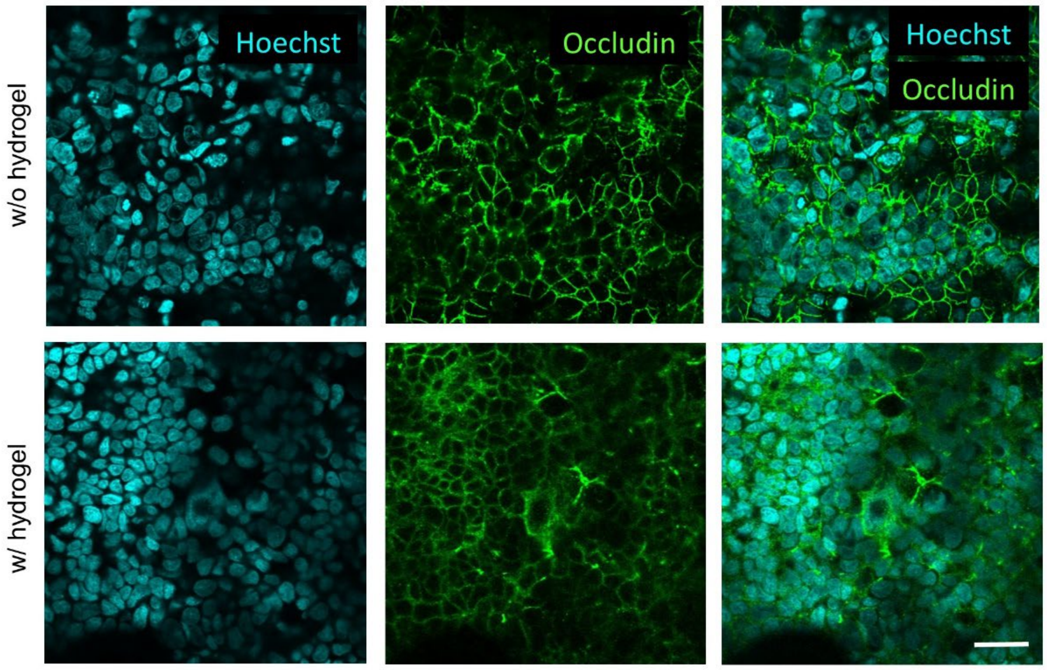

Figure S5. Immunofluorescent analysis of the cell nuclei (Hoechst) and qualitative occludin expression on Caco-2 cells cultured in presence of a 3D alginate-based mucus model showing layer integrity on both experimental conditions. Scale bar: 25 μm.
